# ^123^I metaiodobenzylguanidine (MIBG) uptake predicts early relapse of neuroblastoma using semi-quantitative SPECT/CT analysis

**DOI:** 10.1007/s12149-021-01595-7

**Published:** 2021-02-14

**Authors:** Yoshiyuki Kitamura, Shingo Baba, Takuro Isoda, Yasuhiro Maruoka, Masayuki Sasaki, Takeshi Kamitani, Yuhki Koga, Naonori Kawakubo, Toshiharu Matsuura, Kousei Ishigami

**Affiliations:** 1 Department of Clinical Radiology, Kyushu University Hospital, 3-1-1 Maidashi, Higashi-ku, Fukuoka-shi, Fukuoka, 812-8582 Japan; 2 Department of Health Sciences, Graduate School of Medical Sciences, Kyushu University, 3-1-1 Maidashi, Higashi-ku, Fukuoka-shi, Fukuoka, 812-8582 Japan; 3 Department of Pediatrics, Graduate School of Medical Sciences, Kyushu University, 3-1-1 Maidashi, Higashi-ku, Fukuoka-shi, Fukuoka, 812-8582 Japan; 4 Department of Pediatric Surgery, Graduate School of Medical Sciences, Kyushu University, 3-1-1 Maidashi, Higashi-ku, Fukuoka-shi, Fukuoka, 812-8582 Japan

**Keywords:** ^123^I MIBG, SPECT/CT, Semi-quantitative analysis, Neuroblastoma, Pediatric

## Abstract

**Objective:**

^123^I metaiodobenzylguanidine (MIBG) scintigraphy is a useful tool for the diagnosis of neuroblastoma (NB). MIBG uptake is correlated with norepinephrine transporter expression; hence, it is expected that high-MIBG tumors would be more highly differentiated and have a better prognosis than those with lower expression. We have introduced a method of assessing MIBG accumulation semi-quantitatively using SPECT/CT fusion images. The purpose of this study was to evaluate the relationship of ^123^I MIBG uptake measured by semi-quantitative values of SPECT/CT and early relapse of NB.

**Methods:**

We studied the cases of 11 patients (5 males and 6 females, age 5–65 months, median age 20 months) with histopathologically proven NB between April 2010 and March 2015. The early-relapse group was defined as patients who had relapsed within 3 years after the first ^123^I MIBG SPECT/CT exam. Other patients were classified as the delay-relapse group. Uptake of MIBG was evaluated using the count ratio of tumor and muscles. T/Mmax and T/Mmean were defined as follows: T/Mmax = max count of tumor/max count of muscle, T/Mmean = mean count of tumor/mean count of muscle.

**Results:**

The average T/Mmean values of the early-relapse group and delay-relapse group were 2.65 ± 0.58 and 7.66 ± 2.68, respectively. The T/Mmean values of the early-relapse group were significantly lower than those of delay-relapse group (*p* < 0.05). The average T/Mmax of the early-relapse group and delay-relapse group were 8.86 ± 3.22 and 16.20 ± 1.97, respectively. There was no significant difference in T/Mmax values between the two groups.

**Conclusions:**

Low ^123^I MIBG uptake using semi-quantitative SPECT/CT analysis was correlated with early relapse of NB.

## Introduction

Neuroblastoma (NB) is one of the most common but very rare solid malignant tumors of early childhood and occurs in approximately 150 children each year in Japan [1]. NB is derived from primitive neural crest cells found in several areas of the body and is the most common extracranial solid tumor in children age 5 or younger [2].

^123^I metaiodobenzylguanidine (MIBG) scintigraphy is a useful tool for the diagnosis of NB [3]. Hybrid single-photon emission computed tomography/computed tomography (SPECT/CT) is expected to provide additional anatomical information, and more efficient diagnostic capability [4].

The scoring method due to the distribution of bone metastases has been proposed and used as a prognostic method for predicting prognosis using ^123^I MIBG planar image of NB [5, 6]. On the other hand, it is considered that it is difficult to predict prognosis from visual evaluation of planar images or SPECT images of ^123^I MIBG examination [7, 8].

Interpretation of ^123^I MIBG planar images and SPECT images is usually qualitative. Several semi-quantitative scoring systems for ^123^I MIBG examination of NB have been proposed and explored; however, systems based on planar imaging findings and SPECT findings are not typically included [5, 9–15]. Fred et al. [8] reported that the liver-to-tumor count ratio of ^123^I MIBG SPECT images was useful for differentiating NB from other neuroblastic tumors. Although the liver is commonly used as reference organ, MIBG tends to accumulate in the liver because it is metabolized there. In addition, liver uptake of MIBG is not homogeneous; large differences between the right and left lobes have been reported [16], and it is difficult to designate an appropriate region of interest (ROI) or volume of interest (VOI) as the reference. For these reasons, the liver is not an appropriate reference site for analysis when using MIBG.

In the present study, we introduced a new index that uses muscle as a reference. ^123^I MIBG accumulation of muscle is higher than the background, but relatively small because it is not metabolized in muscle. Because of this relatively low accumulation in the muscle, it is difficult to analyze using conventional planar images; not until the more recent development of SPECT/CT fusion images was this technique feasible.

The purpose of this study is to evaluate the ability of the new semi-quantitative values of ^123^I MIBG SPECT/CT to predict early-relapse lesions of NB.

## Materials and methods

### Patients

Inclusion criteria were histopathologically proven neuroblastoma, a maximum interval of 50 days between ^123^I MIBG SPECT/CT examination and surgical intervention and no tumor-specific treatment before the time of scintigraphy. ^123^I MIBG SPECT/CT examinations were performed between April 2010 and March 2015 in 11 consecutive pediatric patients meeting our criteria (Table1). The group consisted of 5 males and 6 females (age 5–65 months, median age 20 months). At the time of diagnosis patients were staged according to the International Neuroblastoma Staging System (INSS) [17] and International Neuroblastoma Risk Group (INRG) risk classification [5]. According to the International Neuroblastoma Pathological Classification (INPC) of NBs, almost all NBs in our study were subtypes of poor differentiation. MYCN amplification was proven by histopathological study. Vanillyl mandelic acid (VMA), homovanillic acid (HVA) and neuron-specific enolase (NSE) before treatment were also measured. Patients were followed up after MIBG examination (270–3016 days, median 1749 days). Treatments for patients were selected based on the clinical staging (surgical resection: 6, chemotherapy: 10, auto peripheral blood stem cell transplantation: 5, radiotherapy: 3). We defined the early-relapse group as patients who had relapsed within 3 years after the first ^123^I MIBG SPECT/CT exam. We defined the date of relapse as when the pediatrician comprehensively diagnosed recurrence based on clinical findings, histological examination, imaging studies and other information. Other patients were classified as the delay-relapse group. This study was approved by the Institutional Review Board of our hospital.

**Table 1:** Patient characteristics

No.	Age (month)	Sex	Body weight (kg)	BSA (m^2^)	Dose (MBq)	MYCN amplification	INSS grade	INRG risk classification	Metastatic lesion	Treatments
1	13	M	7.5	0.37	63	(−)	1	High		Op
2	4	F	6.8	0.32	56	(−)	4 s	Low		CTx
3	18	F	10.9	0.47	81	(−)	3	Med		CTx
4	35	M	15.0	0.62	107	(+)	4	High		Op, CTx, PBSCT
5	22	M	12.0	0.51	88	(+)	4	High		Op, CTx, PBSCT, RTx
6	65	F	13.5	0.61	106	(+)	4	High		CTx, PBSCT
7	56	F	15.6	0.67	116	(−)	3	High	Liver, bone	CTx, PBSCT, RTx
8	11	F	9.2	0.41	72	(−)	4	Med		Op, CTx
9	8	F	6.8	0.32	55	(−)	4	Med	Liver, bone	CTx
10	31	M	11.3	0.49	86	(−)	4	High	Liver, bone	Op, CTx, PBSCT, RTx
11	20	M	10.7	0.47	81	(−)	4s	Low	Bone	Op, CTx

*BW* body weight, *BSA* body surface area, *INSS* International Neuroblastoma Staging System, *INRG* International Neuroblastoma Risk Group, Op surgical treatment,* CTx* chemotherapy, *RTx* radiotherapy, *PBSCT* peripheral blood stem cell transplantation

### Acquisition protocol

^123^I MIBG SPECT/CT examinations were performed under thyroid blockade with sodium perchlorate over 3 days at 5 ml per day before administration. ^123^I MIBG was injected intravenously with a body-weight-appropriate dose (55–116 MBq). A SPECT/CT system (Symbia T6, Siemens Medical Solutions, Knoxville, TN, USA) was used for imaging, which was started 24 h after administration of ^123^I MIBG.

The whole-body imaging protocol consisted of spot images of the whole-body dorsal/ventral view in a matrix of 256 × 256 pixels. The whole-body scan was obtained at a table feed rate of 7 cm/min.

SPECT images of the abdomen were available in all patients, with acquisitions in 50 views (40 s/view) and iterative reconstruction by OSEM in 8 iterations, with 10 subsets for a final matrix of 128 × 128 pixels. Scatter correction with using triple energy window (TEW) method and CT-based attenuation correction was also used for SPECT image reconstruction. To avoid motion artifacts, light sedation was necessary in all patients. CT scans were also performed with a 5-mm section thickness (130 keV, 30 mAs).

### Image analysis

**Fig. 1:**
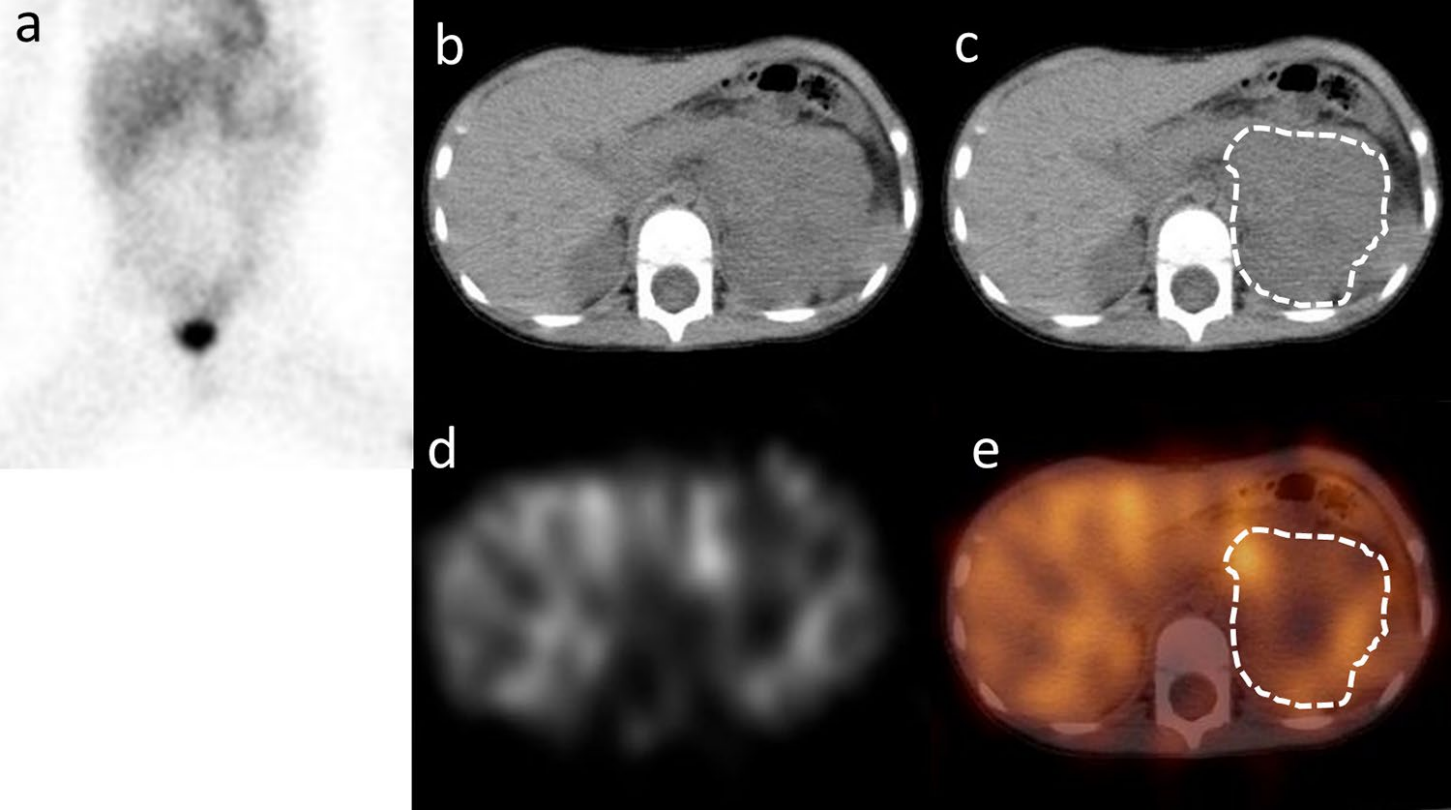
Planar (**a**), CT (**b**, **c**), SPECT (**d**) and SPECT/CT fusion image (**e**) of 35-month-old male (case No. 4). Planar image was anterior image of body lesion. The region surrounded with a dotted line of the CT image (**c**) and the SPECT/CT fusion image (**e**) is designated primary tumor lesion. Approximate T/Mmean was 3.30. MYCN amplification was positive in the histopathological study. He had surgical treatment, chemotherapy and autologous peripheral blood stem cell transplantation; unfortunately, he relapsed 398 days after this scintigraphy examination and died on day 454

**Fig. 2:**
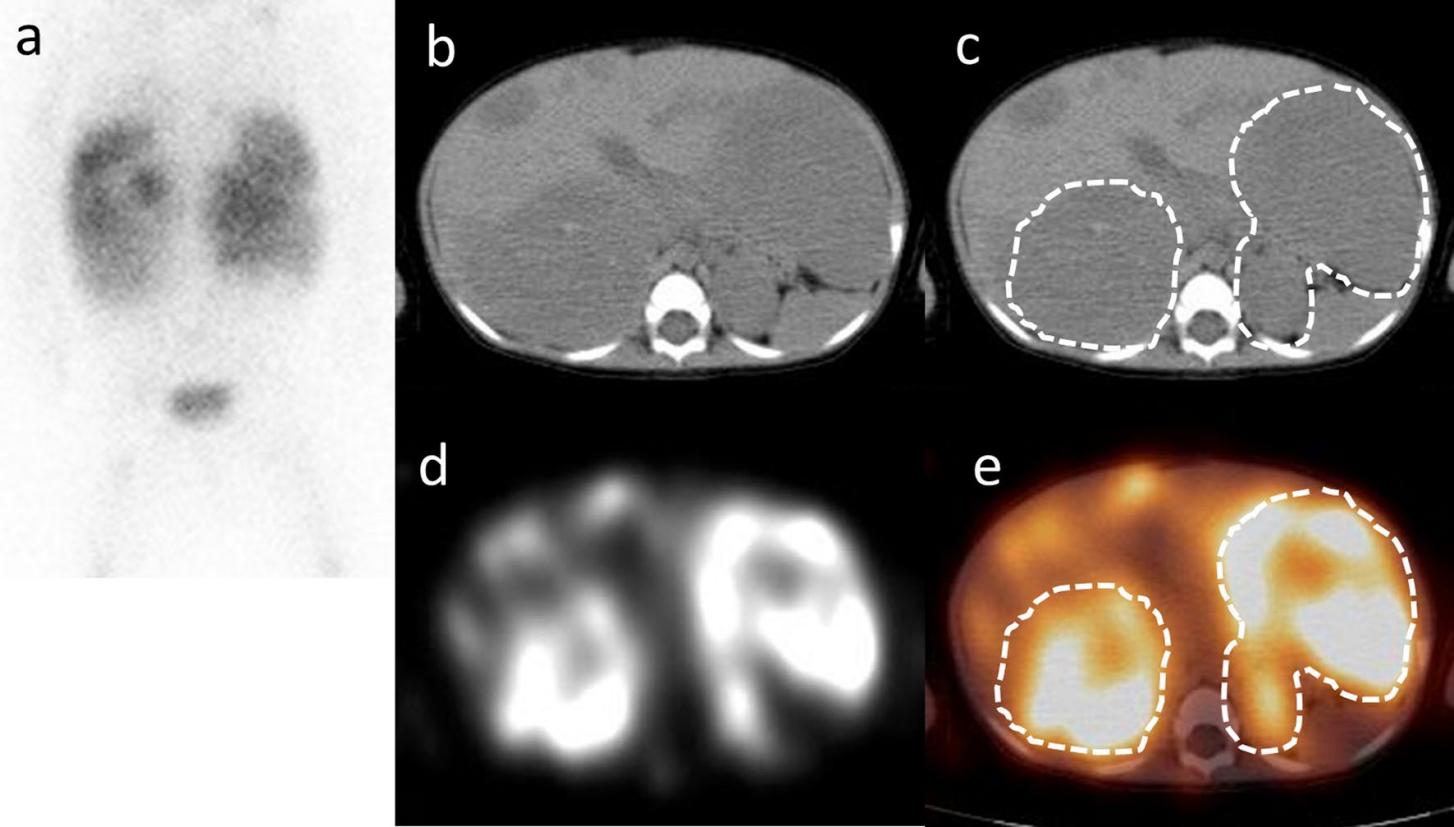
Planar (**a**), CT (**b**, **c**), SPECT (**d**) and SPECT/CT fusion image (**e**) of an 11-month-old female (case No. 8). Planar image was anterior image of body lesion. The region surrounded with a dotted line of the CT image (**c**) and the SPECT/CT fusion image (**e**) is designated the primary tumor lesion. The T/Mmean was approximated as 6.85. There was no MYCN amplification in the histopathological study. She received surgical resection after chemotherapy and remained relapse-free for 5 years after this scintigraphy examination

**Fig. 3:**
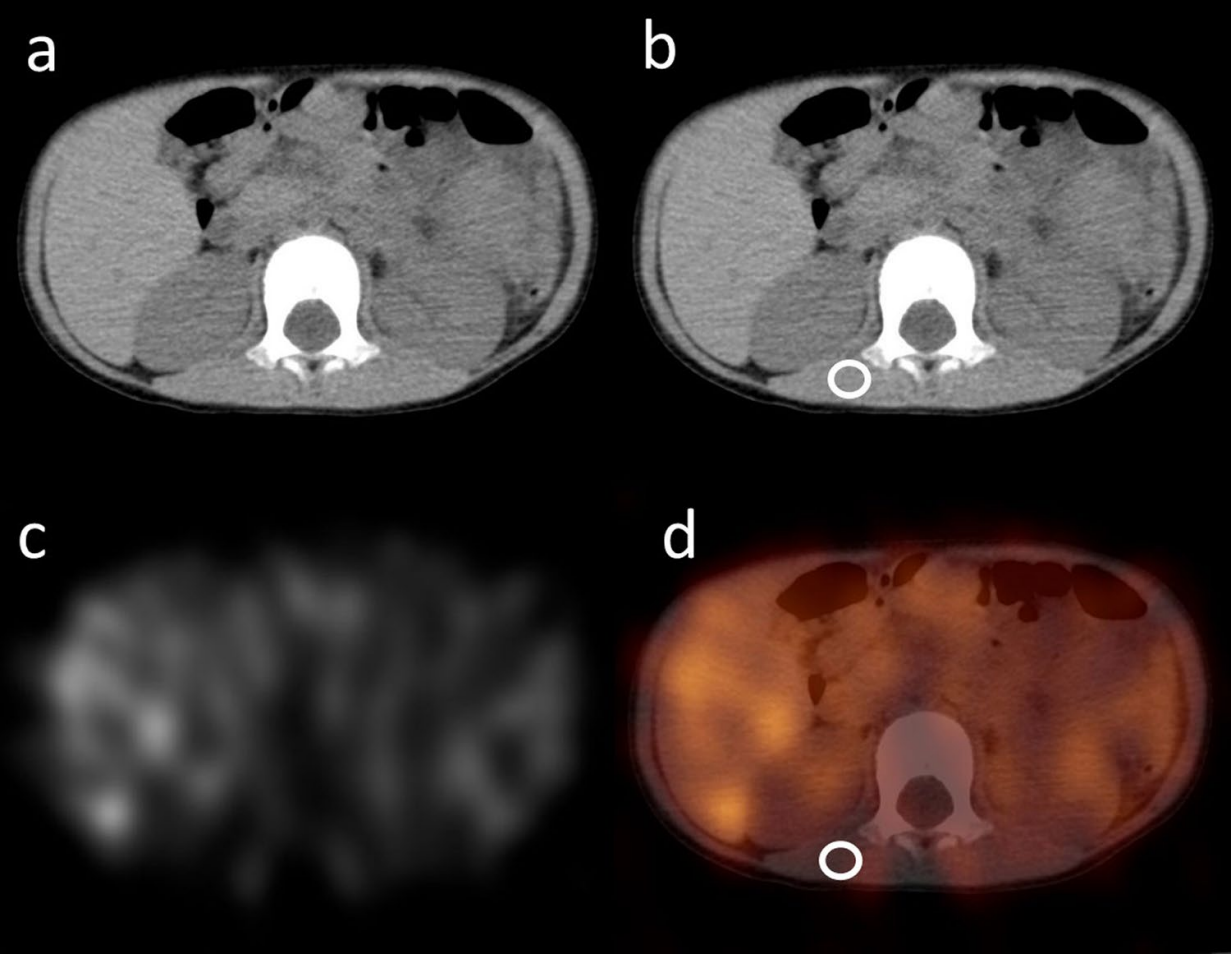
CT (**a**, **b**), SPECT (**c**) and SPECT/CT fusion image (**d**) of case No. 4. For the VOI of muscle in this case, 1-cm-diameter sphere of VOI was settled in the second vertebrae level of the right latissimus dorsi muscle (**b**, **d**; circled area)

Individual data analyses were performed by two experienced observers (Y.K. and S.B.). ^123^I MIBG SPECT/CT images were reviewed on a SYNAPSE workstation (FUJIFILM Medical, Tokyo, Japan). To assess the anatomical site and the exact extent of disease, all patients underwent CT or MRI before or after ^123^I MIBG SPECT/CT with a mean interval of 5 days. The VOI of the primary tumor lesion was determined by consensus of two observers (Figs. 1, 2). VOI of muscle was designated individually in each patient using a 1-cm-diameter sphere of the latissimus dorsi muscle (Fig. 3). These VOIs were designated using an IntelliSpace Portal 6.0 workstation (Koninklijke Philips N.V., Amsterdam, The Netherland). The maximum and mean counts of both tumor and muscle VOIs were noted. The mean CT Hounsfield unit value of each tumor was also measured. The tumor-to-muscle ratios using the max counts and mean counts of VOIs (T/Mmax and T/Mmean) were calculated as semi-quantitative parameters using the following formulas:

$${\text{T}}/{\text{Mmax }} = {\text{ max count of tumor }}/{\text{ max count of muscle,}}$$

$${\text{T}}/{\text{Mmean }} = {\text{ mean count of tumor }}/{\text{ mean count of muscle}}{.}$$

### Statistical analysis

Data are presented as single values or as means ± standard errors of the mean unless otherwise specified. Semi-quantitative parameters were compared between the early-relapse and delay-relapse group using Mann–Whitney’s *U *test. Relapse-free survival analysis was performed using cut-off values of the semi-quantitative parameters as determined by ROC analysis. If value of two groups was clearly separated by ROC analysis, cut-off value was settled as minimal integer between two groups. Correlation between semi-quantitative parameter and each other conditions as MYCN amplification, existence of metastases, VMA and HMA was evaluated using Chi-square test or correlation coefficient. *p* values < 0.05 were considered to indicate statistical significance.

All statistical analysis was performed using JMP 12 (SAS Institute Inc., Cary, NC, USA).

## Results

The results of measurements of all 11 patients are given in Table 2. All patients but one survived the entire follow-up period.

**Table 2:** Results of patients

No.	Age (month)	Sex	VMA (mg/g Cr)	HVA (mg/g Cr)	NSE (ng/mL)	Tumor volume (cm^3^)	Mean HU	T/M max	T/M mean	RFS (day)
1	13	M	32.9	20.8	25.5	3.9	50	6.73	6.21	3016
2	4	F	860	650	112.1	816.3	32.9	12.48	9.13	3013
3	18	F	207.4	147	80	151.9	40.5	14.73	13.57	2499
4	35	M	29.7	14.7	152	127.0	38	6.40	3.30	398
5	22	M	7.9	33.6	584.2	357.4	33.8	6.79	2.46	762
6	65	F	14.2	8.1	173.6	27.9	34.5	3.71	2.19	236
7	56	F	93.9	117.3	353.9	645.7	35.6	15.42	6.86	1945
8	11	F	1725	1728	303.5	658.7	36.1	6.25	8.00	1903
9	8	F	621.3	555.6	430.5	1070.4	36.7	6.75	5.50	1749
10	31	M	431.4	165.9	304.3	137.1	40.3	7.95	6.30	1630
11	20	M	72.2	36.5	19.7	28.9	35.3	5.71	5.71	1396

*VMA* vanillyl mandelic acid, *HVA* homovanillic acid, *NSE* neuron-specific enolase, *HU* Hounsfield unit, *RFS* relapse-free survival

**Fig. 4:**
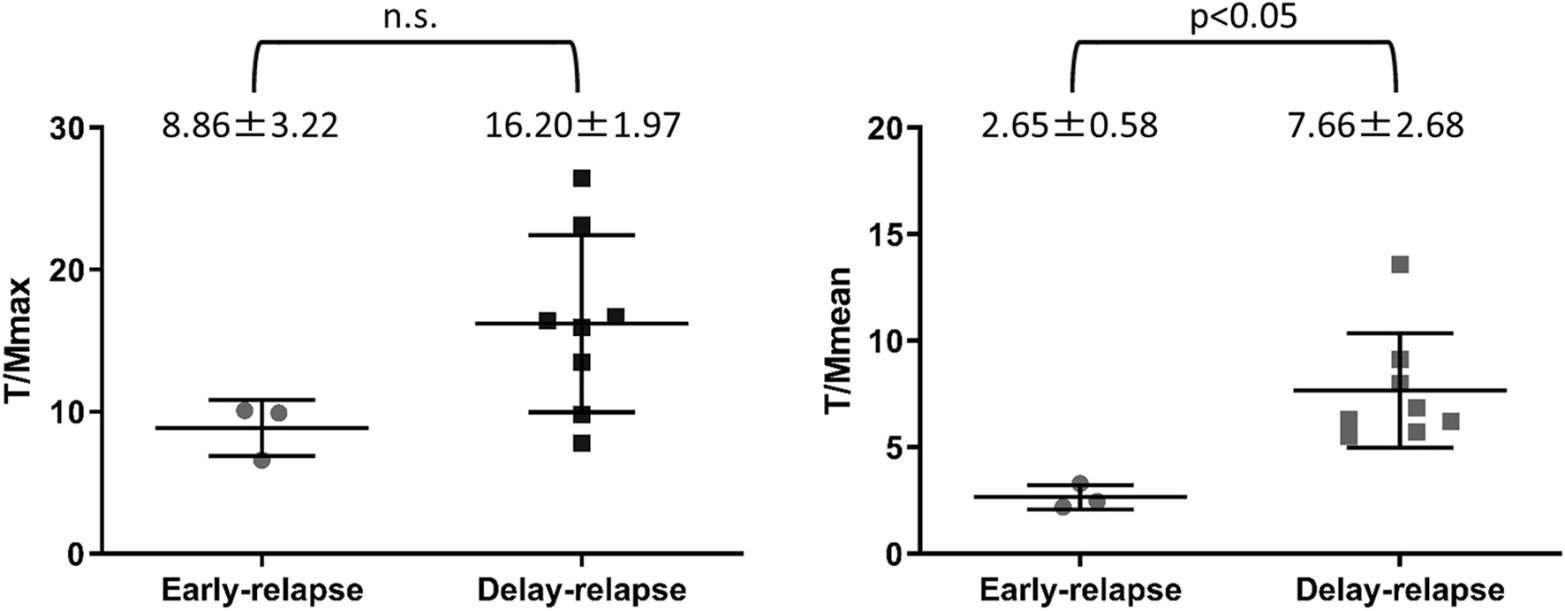
T/Mmax and T/Mmean of the early-relapse and delay-relapse groups. The T/Mmean of the early-relapse group was significantly lower than that of delay-relapse group (*p* < 0.05). There was no significant difference in the T/Mmax values of the two groups

T/Mmax values for the early-relapse and delay-relapse groups were 8.86 ± 3.22 and 16.20 ± 1.97, respectively. There was no significant difference in the T/Mmax between the two groups. T/Mmean values for the early-relapse group and delay-relapse group were 2.65 ± 0.58 and 7.66 ± 2.68, respectively. The T/Mmean value of the early-relapse group was significantly lower than that of the delay-relapse group (*p* < 0.05) (Fig. 4).

**Fig. 5:**
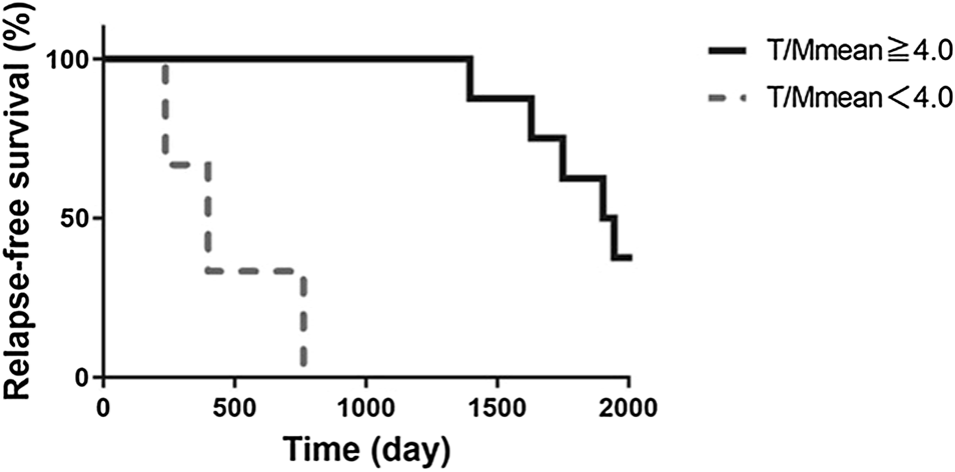
Relapse-free survival analysis of T/Mmean. The survivals of the early-relapse and delay-relapse groups were clearly separated by T/Mmean using a cut-off value of 4.0. The log-rank test showed a statistically significant difference in relapse-free survival between the high- and low-T/Mmean groups, at *p* < 0.01

Relapse-free survival analysis of T/Mmean is shown in Fig. 5. The survivals of the early-relapse and delay-relapse groups were clearly separated using the cut-off value of 4.0. The log-rank test showed a statistically significant difference between the high- and low-T/Mmean groups (*p* < 0.01).

There was a statistically significant relationship between T/Mmean and MYCN amplification using Chi-square test (Table 3; *p* < 0.01). T/Mmean values of all patients with positive MYCN amplification were lower than 4.0.

**Table 3:** 2 × 2 table between T/Mmean and MYCN amplification

	MYCN amplification	
	(+)	(−)
T/Mmean		
≧ 4.0	0	8
< 4.0	3	0

There was no significant relationship between T/Mmean and the presence of distant metastases (Table 4). All cases of the early-relapse group appeared to be free of distant metastases at the time of the MIBG examination.

**Table 4:** 2 × 2 table between T/Mmean and distant metastases

	Distant metastases	
	(+)	(−)
T/Mmean		
≧ 4.0	4	4
< 4.0	0	3

**Fig. 6:**
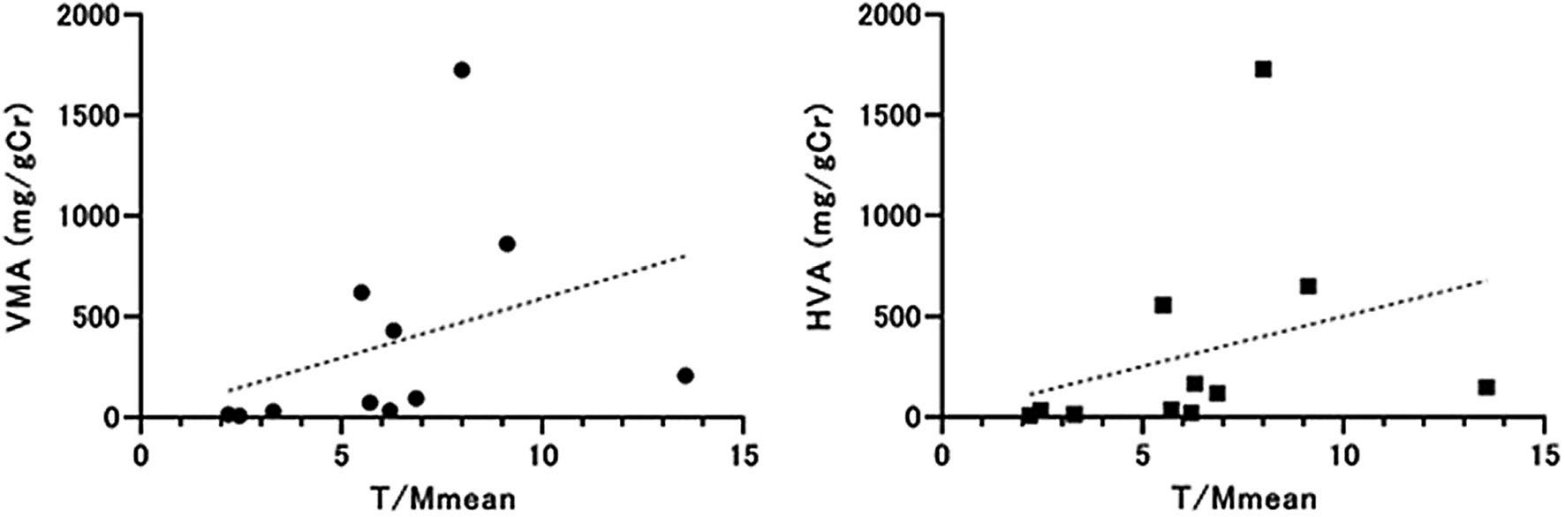
Correlation between T/Mmean and VMA and between T/Mmean and HVA. There was weak positive correlation between VMA and T/Mmean (*R*^2^ = 0.130, *p* = 0.28) and also there was weak positive correlation between VMA and T/Mmean (*R*^2^ = 0.097, *p* = 0.35) with no statistical significance

There was weak positive correlation between VMA and T/Mmean, but it was not statistically significant (*R*^2^ = 0.130, *p* = 0.28). Also, correlation between HVA and T/Mmean was weak with no statistical significance (*R*^2^ = 0.097, *p* = 0.35) (Fig. 6).

## Discussion

Our study introduces the tumor-to-muscle count ratios of T/Mmax and T/Mmean as new semi-quantitative values for ^123^I MIBG SPECT/CT; they represent the ratios of the maximum and mean values of MIBG count, respectively. As a result of our analysis, the T/Mmean ratio of the delay-relapse group was found to be significantly higher than that of early-relapse group. This result may relate with the expression of the MIBG transporter of the tumor. It is thought that the expression of the norepinephrine transporter (NET) and vesicular monoamine transporters 1 and 2 (VMAT1 and VMAT2) are correlated with MIBG avidity in neuroblastoma [18, 19]. Temple et al. [19] reported that the expression of VMAT2 is significantly lower in MYCN-amplified neuroblastomas. All patients of the early-relapsed group had MYCN amplification in this study, which contributed to their low-T/Mmean values.

Tumor necrosis also seemed to be correlated with low T/Mmean, but we did not investigate the exact volume of the necrotic lesion in the tumor. Though CT Hounsfield units may be related with the degree of necrosis in the tumor, there was no statistical difference between the CT Hounsfield units of the tumor of the early-relapse group and those of delay-relapse group (result not shown).

The VMA and HVA of early-relapse group were lower than those of the delay-relapse group and there was weak positive correlation between T/Mmean and VMA or between T/Mmean and HVA. It is known that both VMA and HVA reflect tumor volume and catecholamine metabolism in NB [20]. Especially at VMA, Strenger et al. [21] showed that VMA was lower in the MIBG-negative group than in the MIBG-positive group and Verly et al. [22] reported that VMA was lower in MYCN amplification group than non-amplification group. These reports suggest that weak MIBG accumulation results in low VMA and MYCN amplification, which we believe is consistent with the results of our study.

In this study, we calculated T/Mmax or T/Mmean only for the primary tumor lesion and not for the distant metastatic lesions. We did not measure uptake in distant metastatic lesions because we believe that we may not be able to accurately detect all metastases and that the MIBG avidity and distribution may be different for primary tumors and metastatic lesions. Although the results of this study did not include cases with metastases in the early-relapse group, T/Mmean was lower in the early-relapse group than in the delay-relapse group, regardless of the presence or absence of distant metastases. This suggests that T/Mmean may be a prognostic tool for patients with unknown distant metastasis, even if the presence or absence of distant metastases is unknown. The usefulness of T/Mmax and T/Mmean in distant metastatic lesions remains uncertain.

T/Mmax values of the delay-relapse group tended to be higher than those of early-relapse group, but the difference was not significant. Although T/Mmax may also reflect the catecholamine metabolic activity of the tumor, it overestimates the uptake of MIBG when tumors have a heterogeneous MIBG distribution. This may be the reason why no significant difference was found in the results using T/Mmax. On the other hand, the measurement of T/Mmax has the advantage that the margin of VOI does not have to be carefully considered and is more easily measured. Although not as accurate a predictor as T/Mmean, T/Mmax may have some use in prognostic evaluation.

We decide to use ^123^I MIBG count of muscle for reference lesion. While the accumulation of ^123^I MIBG in the muscle lesions is low, it is higher than the background count and more homogenous than liver lesions. Determining the VOI of a tumor or muscle is difficult with SPECT images alone because the boundaries of the organs are unknown or unclear. It is also difficult to apply the positional information from CT or MRI performed at different examinations. On the other hand, with SPECT/CT, SPECT and CT images are taken almost simultaneously, and the location information of the tumor and muscles are identical. This makes it possible to determine the VOI relatively accurately with SPECT/CT.

This study did not include patients treated with ^131^I MIBG therapy and the usefulness of T/Mmean for predicting the effect of ^131^I MIBG therapy is unclear. It has been reported that the accumulation of ^131^I MIBG is stronger than that of ^123^I MIBG [23], ^131^I MIBG may show good accumulation and therapeutic effect when the T/Mmean is high, but it needs further study.

There were several limitations in this study. First, this study was retrospective. A prospective study is needed to confirm the effectiveness of ^123^I MIBG T/Mmean values at predicting relapse. Second, the small number of patients limited our analysis. This is due to the fact that NB is a very rare tumor. However, our results show a clear difference in T/Mmean between the early-relapse group and the delay-relapse group. Therefore, we can strongly expect T/Mmean to be a good prognostic indicator for treatment prognosis and we think if the 123I MIBG T/Mmean of pre-treatment NB is low, more careful post-treatment follow-up of the disease may be required, whether or not metastatic lesions are present.

## Conclusions

In conclusion, we found that a low level of ^123^I MIBG uptake using semi-quantitative analysis of SPECT/CT was correlated with early relapse of neuroblastoma. This parameter shows promise as a predictor of treatment response in patients with neuroblastoma.
